# Testing an Innovative Gait Training Program in Immersive Virtual Reality for Healthy Older Adults: Protocol for a Randomized Controlled Trial

**DOI:** 10.2196/57866

**Published:** 2025-05-21

**Authors:** Nicolas Mascret, Lisa Delbes, Cédric Goulon, Gilles Montagne

**Affiliations:** 1 Aix-Marseille Univ, CNRS, ISM Marseille France

**Keywords:** virtual reality, VR, exercise, locomotion, adaptive behavior, aging, accident prevention, falls

## Abstract

**Background:**

Impaired gait adaptability is one of the major causes of falls among older adults owing to inappropriate gait adjustments in cluttered environments. Training programs designed to improve gait adaptability behavior in a systemic approach may prevent falls in older adults. Recently, virtual reality (VR) technology has been prominent as a relevant gait training tool because of its training implementation potential.

**Objective:**

This study was designed to compare the effectiveness of a VR-based gait training program (VR group) for improving gait adaptability behavior and, thus, reducing the risk of falls relative to a conventional training program such as Nordic walking (NW; NW group). We hypothesized that the VR-based gait training program will lead to greater gait adaptability improvements.

**Methods:**

We will be conducting a randomized controlled trial with pretests, posttests, retention tests, and follow-up. In total, 40 healthy independent-living community dwellers (aged between 65 and 80 years) will be allocated, after a general medical examination, to the VR or the NW group for a training program of 6 weeks. Primary outcomes related to gait adaptability capacities (ie, analysis of adjustments made in different locomotor tasks) and acceptance of the VR device (ie, analysis of acceptance) will be assessed before and after the intervention and 1 month after the completion of the training program (retention). A follow-up will be done during the 12 months after the completion of the gait training program.

**Results:**

Data collection will begin in September 2025, and the first results are expected in December 2025.

**Conclusions:**

The findings of this study may demonstrate the relative relevance of a gait training program in VR versus a conventional one for improving gait adaptability behavior in healthy older adults and, thus, prevent the chances of a fall.

**International Registered Report Identifier (IRRID):**

PRR1-10.2196/57866

## Introduction

### Fall Prevention in Older Adults

Falls are a significant health issue because they lead to severe injuries, hospitalizations, and death [1,2]. They also lead to serious physical and psychological restrictions after the fall, with a decline in social activities and a reduced quality of life [3]. Repeated falls often occur after a first fall [4]. Given that fall history is the strongest predictor of future falls [5], gait training programs must also be offered to healthy older adults with no fall history. Therefore, these fall prevention programs must be developed to avoid (or at least delay) the first fall occurrence leading to potential, future repeated falls.

Impaired gait adaptability, which is the ability to adjust locomotion parameters to move safely in cluttered environments, is one of the major causes of falls among older adults [6,7]. In cluttered environments, inappropriate gait adjustments can cause older people to hit obstacles or distribute stride length adjustments less than optimally and thus cause falls [2,8,9]. Some studies have shown that impaired gait adaptability is associated with a high risk of falls in older adults [10-12]. To prevent falls in older adults, training programs, including physical activity, designed to improve gait adaptability are thus particularly relevant [13,14].

In fall prevention, several studies have shown the beneficial effects of physical activity, focusing either on one specific (analytic approach) or multiple (systemic approach) physical components [15-18]. In this study, we decided to focus on training programs with a systemic approach, which have been shown to lead to greater improvement of both functional capability and quality of life compared with those based on an analytic approach [19,20].

### Gait Training Programs Adopting a Systemic Approach to Prevent Falls

Training programs adopting a systemic approach can be provided by conventional gait training (without the use of dedicated technologies) or by gait training using new technologies.

#### Conventional Gait Training

In conventional gait training, Nordic walking (NW), defined as “Scandinavian walking with poles,” appears, for instance, as a relevant physical activity in the fall prevention context. This type of walking training promotes an easily accessible physical activity that can be performed by many people to improve their gait capacities [21]. Recent systematic reviews showed that NW training leads to beneficial effects on many biomechanical parameters and motor skills development [22] and appears as a promising and effective way to improve motor capacities in older adults [23], such as increases in arm-leg coordination [24], gait speed and step length [25], and postural control and gait parameters [26-28]. Gait improvements obtained by NW training also reduce the risk of falls [29]. However, in recent years, the use of new technologies has given new impetus to the development of gait training programs based on a systemic approach [30].

#### Extended Reality at the Service of Gait Training

Among these new technologies, extended reality (XR) is an emerging technology that involves virtual reality (VR), augmented reality (AR), and mixed reality [31]. VR is defined as a computer-simulated environment that includes real-time simulation and interaction through multiple sensory channels and a sense of being physically and psychologically present in another place by completely occluding the real world [31]. Contrary to VR, the real world is not occluded in AR: virtual objects are overlaid in the real-world environment. In the same way as in AR, in mixed reality, virtual objects are overlaid in the real-world environment; however, users can physically interact with these virtual objects.

Several studies have demonstrated the effectiveness of XR gait training on balance and gait ability in older adults [32-37]. Shema et al [36] conducted a 5-week VR gait training program for older adults with a fall history. Participants walked on a treadmill and performed obstacle negotiations during a virtual course, while their displacement was displayed in a virtual environment (VE) represented on a screen located in front of them. In their study, Mirelman et al [35] provided a 6-week VR gait training program for older adults with a high risk of falls. During the training sessions, participants walked on a treadmill and performed several goal-directed locomotion tasks (ie, multiple obstacles and paths either with or without distractors), while their displacement was displayed in a VE represented on a screen located in front of them. For their part, van Ooijen et al [37] provided a 6-week AR gait training program for older adults with fall-related hip fractures. Participants walked on an instrumented treadmill on which a visual context was directly displayed on the belt, proposing various goal-directed locomotion tasks (eg, stepping and obstacle avoidance).

These XR gait training programs led to greater improvements in gait ability and thus a reduction in the risk of falls compared with conventional ones [35-37]. However, the implementation of these programs may have some limitations in terms of gait training relevance. Consequently, some precautions need to be considered to identify areas for improvement of XR gait training programs.

#### Four Precautions for XR Gait Training Program Implementation

##### Precaution 1: Preserving the Ecological Validity of the XR Setup

The actual use of these XR devices may be examined to ensure the ecological validity during gait adaptability training programs [38] in terms of the level of immersion and locomotion.

First, the level of immersion is an important characteristic of XR devices in training implementation [39] because a high level of immersion leads to high engagement and enjoyment [40]. The XR setups used in previous studies did not make it possible to provide a high level of immersion. Shema et al [36] and Mirelman et al [35] used the same VR setup, which provided a third-person view of the foot avatar in the VE presented on a small screen in front of the participants, leading to a low level of immersion. An immersive experience with a first-person view (ie, the transposition of the usual user’s viewpoint into the VE) seems primordial in a gait training program intended to improve gait adaptability capacities. In their review, Liu et al [38] demonstrated that the use of fully immersive XR devices with older adults induced greater gait improvements compared with nonimmersive ones. Future XR gait training program implementation with older adults should be based on the use of a fully immersive XR device (as a VR head-mounted display [VR-HMD]).

Second, in the 3 previous studies, older adults walked on a treadmill. This constitutes a second limitation in gait training implementation, given that the use of a treadmill gives rise to partly different gait kinematics compared with natural locomotion (eg, sagittal plane kinematic differences at foot strike [41]). Conversely, overground locomotion preserves natural locomotion, allowing a high level of ecological validity and natural interaction with virtual objects [42,43].

A recent study underlined the high ecological validity of a VR setup that combines a fully immersive experience and overground locomotion. Delbes et al [44] aimed to compare the gait behavior of older adults during a similar goal-directed locomotion task (ie, a simple locomotor pointing task) performed in the real world and in VR. In the 2 conditions, participants walked and performed the task with overground locomotion. In the VR condition, participants wore a VR-HMD displaying 2 VEs: a replica of the real one and a new environment. The authors demonstrated the similarity of the gait adaptability behavior produced in the real world and in VR. The use of a VR-HMD coupled with overground locomotion appears particularly relevant for future XR gait training program implementation by (1) ensuring a first-person view with a high level of immersion, (2) ensuring nonconstraint locomotion, and (3) proposing enjoyable scenarios that mimic real-life tasks.

##### Precaution 2: Following Motor Learning Principles

According to Hendry et al [45], gait adaptability training program implementation should be based on 4 motor learning principles.

The first principle is related to the variability of practice, which is necessary to optimize the improvement of gait adaptability behavior in older adults. Task variability is crucial for learning optimization by confronting older adults with a large range of potential gait adjustments based on their own gait capacities. In previous studies, older adults were confronted with various environmental lighting conditions [36]; changes in the number, size, and shape of obstacles [35]; and configurations of obstacles [37] with a training progression. Future XR gait training implementation should include the manipulation of variability based, for example, on the modulation of VE spatial constraints (obstacle characteristics and configurations).

The second principle is related to the amount of practice and, more precisely, to task repetitions. Performing any repetitions is a *sine qua non* for optimizing any learning [45]. In previous studies, experimenters provide a very global estimation of the amount of practice, for example, 15 sessions of 60 minutes [36], 18 sessions of 45 minutes [35], and 30 sessions of 40 minutes [37]. However, there is no precise information about the number of repetitions for each task performed. The combination of these first 2 principles underlines the importance of the concept of “repetition without repetition” [46,47]. Future XR gait training implementation should be based on a large amount of practice (task repetition) coupled with a large variability of practice.

The third principle is related to the individualization of the training content based on individual characteristics (eg, foot size and leg length) to lead to greater effects of the gait training program [20,48]. In previous studies, task manipulation cannot be regarded as task individualization of the training content because task configurations were manipulated without considering individual characteristics. Mirelman et al [35] manipulated the treadmill speed and the duration of training. However, this kind of task configuration was only based on the modulation of global parameters. Task individualization should also provide a gradual increase in task difficulty based on individual capacities and performance to induce an appropriate level of challenge to optimize motor learning and engagement [45,48]. In summary, future XR gait training implementation should include individualized training content based both on considering the anthropometric characteristics of the participants and on their rate of progress.

The fourth principle is feedback supplementation during the training sessions, which improves motivation, engagement, and adherence and enhances motor learning [45,49]. Various types of visual and auditory feedback, informing about performance and results, were provided during training sessions in previous studies [35-37] and at the end of the sessions in the study by Mirelman et al [35]. Future gait training program implementation should provide feedback (ie, knowledge of performance and result) with a gradual decrease in the frequency of feedback over the training program. Finally, to optimize motor learning, feedback may be requested by the participant to avoid a guidance effect [50].

##### Precaution 3: Choosing the Right Level of Analysis to Characterize the Effect of Training

In previous studies [35-37], the examination of gait behavior was limited to gait parameters reflecting only the participant behavior (eg, gait speed, step width, step length, and cadence). As mentioned previously, an analytic approach (based on the measure of global gait behavior parameters) only allows an overall understanding of the gait behavior of older adults. However, a precise characterization of participant behavior in relation to the environment (ie, the state of the agent environment system) is needed to provide a better understanding of the improvements induced by gait training.

Additional levels of analysis are consequently required to study gait adaptability behavior appropriately during goal-directed locomotion tasks. During obstacle negotiation, the evolution of the state of the agent environment system can be investigated through intertrial and trial-by-trial analyses, which allow gait adjustment strategies to be characterized in relation to obstacles (eg, the modulation of each footfall target distance [and its variability] over the obstacle approach). Gait adjustments are indeed based on a continuous coupling between information and movement [51]. These additional levels of analysis allow us to investigate the agent environment system as a whole and not the agent alone. Finally, the study of global gait behavior (as done in previous studies) coupled with the study of gait adaptability behavior (ie, based on gait adjustment indicators of the state of the agent environment system) constitutes complementary levels of analysis to characterize gait behavior in older adults more precisely.

##### Precaution 4: Studying the Acceptance of the XR Device Used

Merely examining the effectiveness of a training program is not sufficient. Acceptance of the XR device should also be investigated because attitudes of older adults toward the technology used in the training program may influence its effectiveness [52,53]. The fact that the use of an XR device is objectively effective does not mean that it is necessarily accepted, and, conversely, an XR device that is accepted is not necessarily effective. However, in the studies by Mirelman et al [35], Shema et al [36], and van Ooijen et al [37], only the effectiveness of XR gait training programs was proven without considering the acceptance of the XR device by older adults.

Some studies have investigated the acceptance of XR devices in older adults, for instance, acceptance before the first use of a VR-HMD intended to prevent falls [53]. This study revealed that older adults’ intention to use the VR-HMD was positively predicted by perceived usefulness, perceived ease of use, and perceived enjoyment and that no initial blockages appeared among older adults before the first use. Moreover, Delbes et al [44] compared acceptance before and after the use of a VR-HMD specifically intended to prevent falls among older adults. They highlighted that older adults did not intend to use the VR-HMD before the first use, but they did not refuse it either (ie, neutral attitudes toward VR-HMD before the first exposure). Finally, they intended to use it after the first effective use during a single session in VR.

To date, no study has shown the potential evolution of acceptance of the VR-HMD by older adults throughout a gait training program intended to prevent falls. As the study of the effectiveness of an XR gait training program cannot be done without ensuring that the XR device is accepted [52], the effectiveness of the program and the acceptance of the device will be conjointly investigated, in contrast to previous studies.

### Objectives of the Study and Hypotheses

The ambition of our study is to investigate both the effectiveness of a VR gait training program (using a VR-HMD) and the acceptance of the VR device among healthy older adults. The effectiveness of the VR gait training program will be compared with that of a conventional training program of NW. The choice to compare a VR gait training program method to NW reflects the ambition of this work. From our point of view, NW offers the ecological context that is most conducive to the maintenance and even improvement of the functional relationships between perception and action. The walker must constantly adjust locomotion parameters in reference to obstacles to produce adaptive behavior in cluttered environments. Our VR gait training program aims to be the counterpart of NW in a perfectly controlled context thanks to VR technologies. Demonstrating the potential of our protocol would seem more convincing to us if it were established that the improvements induced by using a VR device were equivalent or even superior to those induced *via* NW. The comparison of the gait training programs’ effectiveness will be based on a pre- and postintervention comparison of gait adaptability capacity improvements based on further levels of analyses (as presented earlier). The 2 gait training programs will be offered to healthy older adults (with no fall history) with a systemic approach to fall prevention intended to avoid or at least delay the first fall occurrence. The study of VR-HMD acceptance will be based on the Technology Acceptance Model (TAM) [54-56].

The VR gait training program will be implemented following the different guidelines presented earlier. On the basis of all the previous precautions, our future gait training program in fully immersive VR may potentially improve both functional and psychological outcomes. We hypothesize that both gait training programs will lead to improvements in gait adaptability behavior capacities (hypothesis 1) but that the VR gait training program will induce greater improvements in gait adaptability capacities in comparison with the NW training (hypothesis 2). Finally, we hypothesize that older adults will accept the VR-HMD before the VR gait training program (hypothesis 3) but that acceptance will increase throughout the gait training program (hypothesis 4).

### Study of Secondary Variables

In addition, the study of secondary variables will allow us to compare our results with those of previous studies and provide additional knowledge in the specific population of healthy older adults with no fall history. As done in previous studies [35-37], some specific tests will be carried out before and after the intervention. First, in terms of functional capacities, we will evaluate mobility and balance capacities, which are linked to the risk of falls in older adults [57-59]. Second, in terms of psychological states, we will study motivation toward health-oriented physical activity [60], intrinsic motivation [61], and self-reported fear of falling [62,63], which may impact the risk of falls in older adults. The secondary variables will allow us to investigate the training program’s potential effects on some specific health components, given that interactions exist among gait behavior, functional capacities, and psychological states [64]. Recently, Dermody et al [43] reviewed the high potential of VR-HMD to improve both functional and psychological outcomes in older adults. Finally, in addition to the study of gait adaptability behavior and the acceptance of the XR device, the study of secondary variables will allow us to demonstrate potential general health changes after the gait training program.

## Methods

### Participant Recruitment

The initial recruitment will be carried out from an existing cohort of healthy older adults recruited by the Active Aging Chair.

### Inclusion Criteria and Informed Consent

Participants are independent-living community dwellers aged between 65 and 80 years. They are active older adults with weekly moderate to vigorous physical activity. This study is a part of a broader research program aimed at determining the extent to which new technologies (eg, exergames and XR) can be used to promote healthy aging among active older adults [23,24,65]. Of course, populations of more fragile older adults (eg, older adults who fall) could benefit from an appropriate intervention approach; however, our objective in this study consists, rather, within the framework of a preventive approach of creating the conditions allowing the delay or even avoidance of the occurrence of a first fall.

The exclusion criteria are inability to practice activity at a moderate or vigorous intensity (checked by medical examination supervised by cardiologists), moderate or severe cognitive impairments (ie, a Mini-Mental State Examination score <26 [66]), severe noncorrected visual impairments, and uncontrolled psychiatric disorders.

Every 5 minutes of VR use, the Fast Motion Sickness scale [67], designed to measure cybersickness, will be completed orally by the participant. According to the protocol set up by Keshavarz and Hecht [67], if a participant reaches a score of 15 or more, they will immediately be asked if they wish to stop participating and be told that this will have no harmful consequences for them.

Information about the organization of the gait training programs will be presented to the eligible participants by the principal investigator. Those who are willing to participate will be asked to give informed consent.

### Ethical Considerations

Importantly, this protocol will be submitted during April 2025 to a national ethics committee (ethics committee for research in science and technology of physical and sporting activities) for approval. The approved protocol will be implemented from September 2025 in accordance with the Helsinki Convention. Before the study, participants will give their written informed consent to participate for free in the experiment. For their part, the researchers who carry out the experiment undertake to respect the rules of data confidentiality and the privacy of the participants.

### Study Design

**Figure 1:**
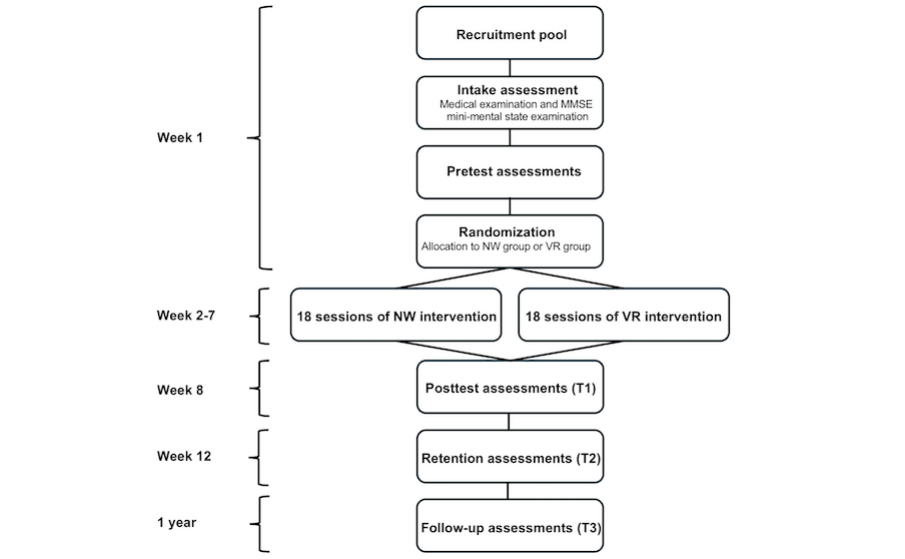
Flowchart of the study design. MMSE: mini-mental state examination; NW: Nordic walking; VR: virtual reality.

This study is based on a comparison of a gait training program in VR (ie, the VR group) with sessions of NW (ie, the NW group). Participants who meet the inclusion criteria will be randomly allocated to the gait training program (in the VR or NW group) using a computer-generated block randomization with a block size of 30 participants (1:1 rate). The data for participants in both groups will be obtained at 3 different times: 1 week before the beginning of the training program (T0), 1 week after completion of the training program (T1), and 1 month after completion of the training program (retention assessments; T2). Moreover, a follow-up (T3) with the incidence of trips and falls will be done during the 12 months after completion of the gait training program. Participants’ flow is presented in Figure 1.

### Gait Training Programs: NW Versus VR

#### Overview

The 2 gait training programs will be dose-matched in terms of duration (ie, 45 min per session) and frequency (ie, 3 sessions per week) of training over a 6-week program (ie, 18 training sessions) following the methodology used in previous gait training studies [35-37]. Previous studies have shown that extensive practice is important for the consolidation and retention of motor memory [68]. Therefore, in line with our ambition to develop long-term improvements in gait adaptability behavior, a large amount of practice in our gait training programs is necessary.

#### NW Group

In the NW group, participants will practice the training sessions in a nature park. They will be instructed to walk with poles continuously around the park. Each training session will consist of a warm-up period (10 min), main exercise (45 min), and cool-down period (5 min). Participants will adapt their locomotion to uneven terrain. They will negotiate natural obstacles such as avoiding a rock or crossing over a tree root, thus improving their gait adaptability capacities. Sessions will be provided by a coach from a multisport club proposing programs of physical activity adapted to older adults. Participants will follow the coach, who will progressively increase the level of difficulty over the 6 weeks, show the way, and adapt to the gait speed.

#### VR Group

**Figure 2:**
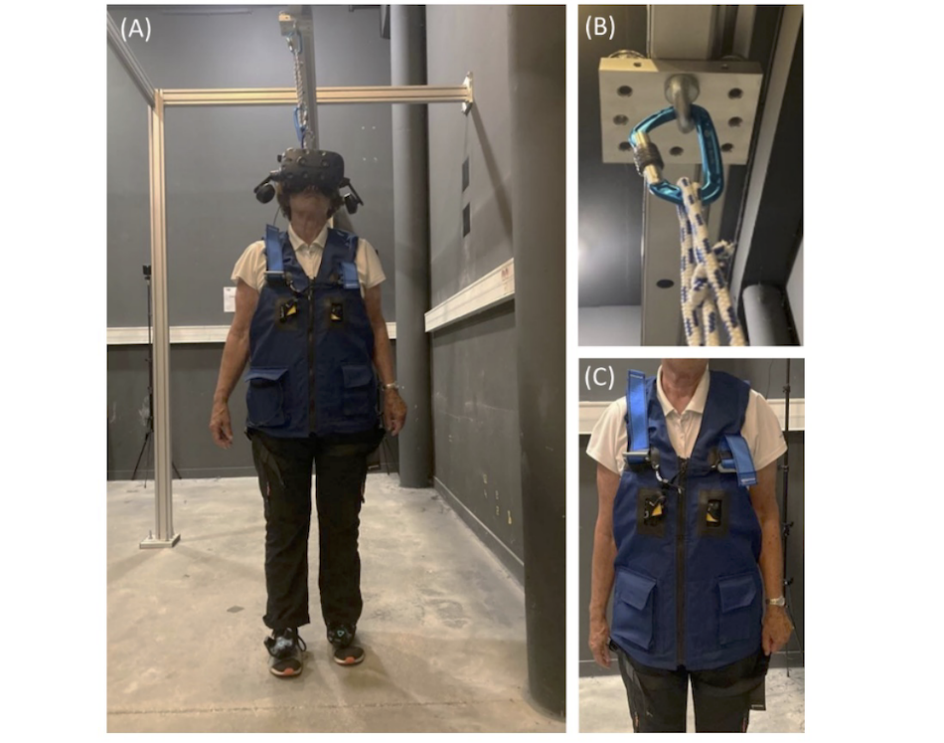
Test equipment. (A) A wireless HTC Vive Pro will be used to display the virtual environment. Two HTC Vive Pro 2.0 trackers will be used to record the positions of the feet and display virtual 3D shoes in the virtual environment; (B) in-ceiling rail; (C) safety harness.

In the VR group, participants will practice the training sessions in an experimental room. In 1 training session, participants will negotiate 60 trials. Sessions will be provided by the principal investigator. Participants will practice gait training sessions in a fully immersive VR. A wireless HTC Vive Pro VR-HMD will be used to display VE (Figure 2A). Participants will wear HTC Vive Pro foot trackers on their feet, which will record their feet positions (in relation to obstacles) and display 2 virtual foot avatars in the VE. A security system consisting of a safety harness (Figure 2C) connected to an in-ceiling rail by a lanyard (Figure 2B) will provide training sessions in a safe context. This setup has been validated in a previous study [44] in which gait adaptability behavior was similar in the real world and in VR.

VR training sessions were created with homemade software developed with Unreal Engine (version 5.1; Epic Games) and OpenXR (Khronos Group). It will be used with a wide panel of AR and VR technologies (Oculus, HTC Vive, HP, etc). The software contains 2 operation modes: design and simulation.

**Figure 3:**
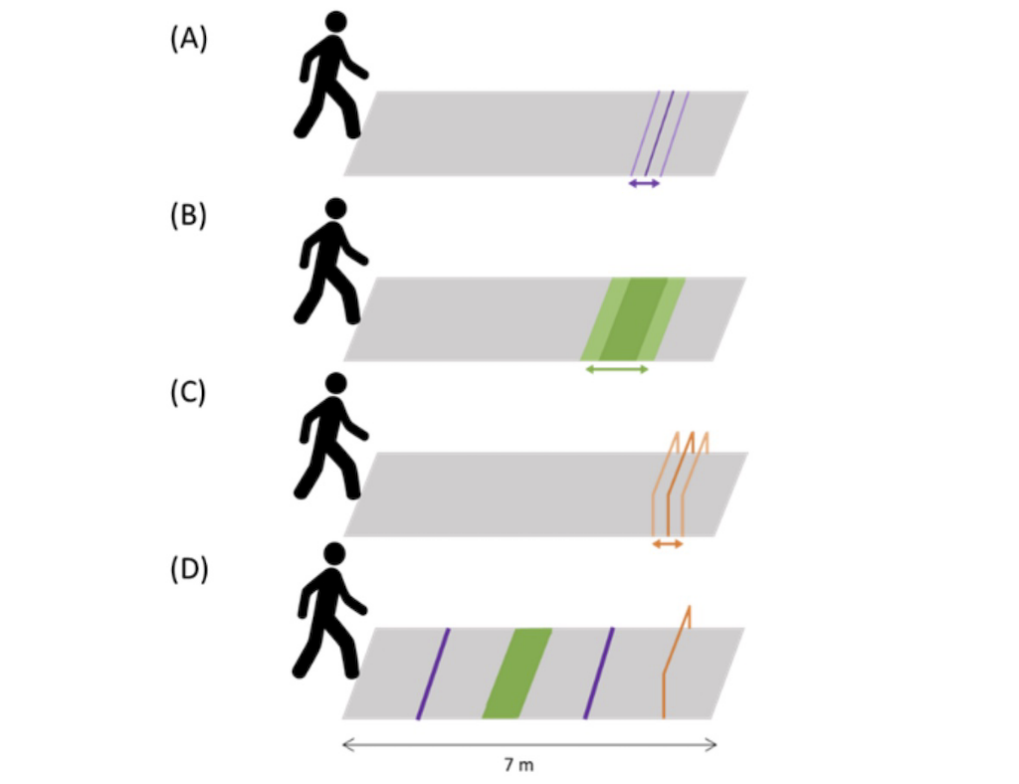
Schematic of the 4 different goal-directed tasks performed on the walkway GaitRite (in gray). (A) simple locomotor pointing task; (B) crossing-over task; (C) stepping-over task; and (D) “mixed condition” with 2 locomotor pointing (in purple), 1 crossing-over (in green), and 1 stepping-over (in orange) tasks.

The design mode is used to create the content of the different training sessions (ie, 60 trials in different configurations per training session) with a user interface. Each kind of task can be modulated *ad infinitum* by changing the position, number, and size of the obstacles. Moreover, configurations will be modulated by the number of different tasks displayed: participants can negotiate one or several kinds of tasks in 1 trial. The 3 tasks mainly used in the training protocols are illustrated in Figure 3. The pointing task consists of positioning the foot as close as possible to the line without exceeding it. The crossing-over task consists of crossing over a horizontal obstacle without meeting it. The stepping-over task consists of stepping over a vertical obstacle without touching it. The technology used (tracker positioned on the feet) makes it possible to locate the feet at any time with reference to virtual obstacles and to determine the performance produced depending on whether the task is carried out in accordance with the success criteria or, conversely, if collisions with obstacles occurred. This method is based on the exclusive use of virtual obstacles. Examples of different trial configurations are provided in Figure 3. In this mode, different criteria of success will be determined at different scales (ie, obstacle, trial, and training session). The criterion of success for each obstacle negotiation is fixed according to the kind of task: pointing accuracy (in cm) for simple locomotor pointing; recovery (in cm) for successive locomotor pointing; and clearance (in cm) for stepping-over, crossing-over, and obstacle avoidance. The criterion of success for each obstacle is set as a certain percentage of successful obstacle negotiation. Finally, the criterion of success for each training session is set as a certain percentage of successful tasks.

The simulation mode is used to display the training sessions created in the design mode and to record participants’ foot positions in relation to obstacles. In this mode, the participant’s anthropometric data are considered to propose individualized session content based on a certain percentage of each participant’s leg length. In real time, the participants’ performance is calculated in terms of obstacle negotiation and completion time. On the basis of these real-time data, the success or failure of the obstacle negotiation will be measured depending on the determined criterion of success. Different types of feedback can be provided to inform the participant about their obstacle negotiation performance. Moreover, at the end of the trial and the training session, additional feedback can be given to inform the participant about their trial and session success, respectively. This feedback can be provided on the screen as text (“success” or “fail”) or coloration of the virtual scene (in green for success or in red for fail).

**Figure 4:**
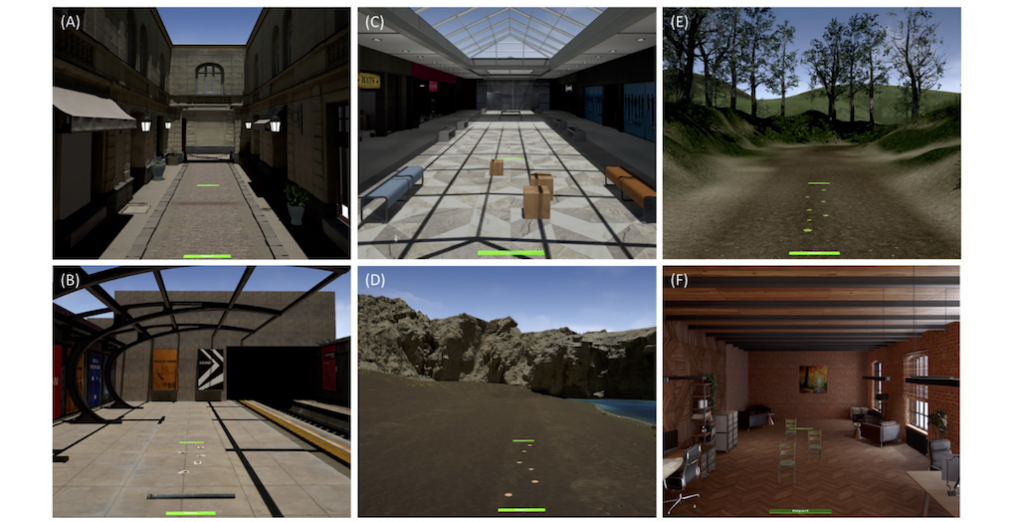
Examples of first-person views of locomotor goal-directed tasks in the different virtual environments (A) cross over road tar in a street, (B) step over a pipe and stepping on successive flyers in a subway, (C) avoid cardboard boxes in a shopping center, (D) step over successive seashells on the beach, and (E) step over successive leaves in a forest, (F) avoid chairs in an office. Green areas represent the start and the finish lines.

Given that the software allows the modulation of the content of the training sessions by setting specific criteria, several goal-directed tasks in various VEs will be provided to propose relevant gait adaptability exercises. Six different VEs have been created (Figure 4): subway, shopping center, forest, beach, office, and street.

Our gait training sessions will be developed with an increasing level of difficulty over the 6 weeks. In terms of progress, 18 levels of difficulty have been created (to be able to provide a new level per new training session). In the first training sessions, only one kind of task will be displayed in a single trial in the presence of a limited number of mainly small obstacles. Progressively, the number and size of the obstacles will increase. Afterward, 2 kinds of tasks will be displayed in a trial. In the last training sessions, participants will negotiate various obstacles with 2 kinds of tasks in a trial. On the basis of the criteria of success previously presented, when a participant succeeds in the training session, they advance to the next level of difficulty for the next training session. Conversely, if they fail, they remain at the same level for the next training session. In addition, the session content has been created with a high level of individualization. As presented earlier, the participant’s anthropometric data will be considered to propose training according to individual capabilities. The obstacle sizes will be proportional to each participant’s leg length. The surface area, the height, and the depth of the obstacle will be modulated, respectively, for the successive locomotor pointing, stepping-over, and crossing-over tasks. Obstacle modulation will lead to equivalent levels of difficulty for all the participants over the training program.

In summary, our software will allow us to implement the main principles of Hendry et al [45] presented in the introduction. The VR training program was designed in particular to optimize the variability of the practice conditions and to allow the learner to have qualitative feedback on the success of the task. The variability of the practice conditions was obtained essentially by manipulating the location of the obstacles, their dimensions, and the nature of the task. The speed of locomotion was not manipulated as such, given that participants were asked for each trial to complete the route as quickly as possible while giving priority to precision. Concerning feedback, at the end of each trial, the participant will have the possibility of requesting feedback related to the knowledge of result (self-controlled feedback). It has been shown that this type of feedback makes it possible to enhance learning by placing the participant at the heart of the learning experience and developing their autonomy [69-71]. One of the main advantages of the self-controlled feedback procedure is that it allows participants to use feedback to optimize control processes without inducing a dependency effect. Indeed, the literature indicates that participants spontaneously implement a fading procedure [71] by requesting feedback massively at the start of the training period and then less often, which protects them from any dependence on feedback [50].

A stratified randomization (with the sex factor) will be conducted, using a computer software program to generate a random sequence. Two strata (male and female) will be identified, and randomization will be applied to each stratum. For instance, when a male older adult participates in the study, he will be the first to be allocated to the male strata, and the group (VR group and NW group) will be determined through randomization applied to the male stratum.

### Outcome Measures and Data Collection

#### Primary Outcome Variables

Two major variables will be computed to investigate the effect of these two gait training programs intended to develop gait adaptability behavior in older adults. In terms of gait adaptability behavior, gait parameters will be computed from an innovative gait adaptability test. In the context of fall prevention, this specific test will allow us to investigate gait adaptability behavior with several levels of analysis. In terms of VR-HMD acceptance, we will examine acceptance before and after the use of the VR device designed to prevent falls in older adults. We will determine attitudes and representations toward the technology device used in this training program to ensure that older adults accept the VR-HMD.

#### Gait Adaptability Test

Gait adaptability capacities will be assessed by a specific gait test created to assess gait parameters during goal-directed tasks. Feet positions will be measured by a 7-m-long portable pressure-sensitive walkway, GaitRite (CIR Systems, Inc), in the real world. The security system (safety harness connected to an in-ceiling rail by a lanyard) will also be used to carry out the tests. First, participants will be instructed to walk at a normal pace on the walkway. The average preferred gait speed, step length, and step duration (with their variabilities) will be calculated over 6 trials. Afterward, physical obstacles will be placed on the walkway. Participants will perform 3 different locomotor tasks: simple locomotor pointing, crossing-over, and stepping-over tasks (Figure 3). They will perform 10 trials of each goal-directed task. Participants will be instructed to negotiate obstacles as quickly as possible without colliding with them. The size of the obstacles will be proportional to the anthropometric data of the participants (ie, based on individual leg length).

For the locomotor pointing task, participants will be instructed to place the right foot tip as close as possible to the lower extremity of the target. The pointing performance (ie, the horizontal distance between the tip and the lower extremity of the target) will be measured. For the crossing-over task, participants will be instructed to cross an obstacle with a depth of 60% of their leg length. The crossing performance (ie, the horizontal distance between the tip and the lower and upper extremities of the target) will be measured. For the stepping-over task, participants will be instructed to step over an obstacle with a height of 20% of their leg length. The stepping performance (ie, the vertical distance between the tip and the target) will be measured using Kinovea Video analysis software (version 0.8.15; 2006-2011 Joan Charmant and Contrib). The position of obstacles will be manipulated (3 different positions each separated by 30 cm intervals) and presented in a randomized order (2 trials for each position condition) to avoid locomotor regularities and prevent any calibration of the distance being covered. Finally, participants will perform 10 trials of a mixed condition: a combination of 2 previous goal-directed tasks (Figure 3).

By recording successive feet positions in relation to the obstacles, gait adaptability behavior will be analyzed through intertrials and trial-by-trial analyses [44,51,72].

In the intertrial analyses, the SD of the toe-obstacle distances and the SD of the step lengths will be measured. If the locomotion behavior produced is functional, the intertrial analysis should reveal inverted patterns of variability [44,51]. This result reveals that step length adjustments are produced to minimize toe-obstacle distance at the last footfall and thus perform optimal foot positioning. We hypothesize that the training carried out should accentuate the presence of compensatory variability (pre- vs posttest comparison) but also that the compensatory variability produced following the training should be more pronounced for the VR group than for the NW group.

The trial-by-trial analysis makes it possible to identify precisely for each trial the presence of adjustments of the step lengths and their distribution throughout the trial by comparing each step length with the average pattern of step length (see the study by Montagne et al [72] for a similar procedure). The second part of the analysis consists in computing for each step the number of adjustments needed to place the foot on the target and the number of adjustments actually implemented [44,72]. Here again, we hypothesized that the training carried out should enable participants to improve their ability to produce adaptive behavior. More precisely, not only should step length adjustments be initiated sooner after training (ie, after the test) so that they can be spread over a larger number of steps but also the adjustments produced should more accurately reflect the adjustments required to succeed in the task. These improvements in adaptive locomotor skills should be more pronounced for the VR group.

To sum up, from this gait adaptability test, we will characterize the gait adaptability behavior with different levels of analysis. In addition to a traditional analysis of locomotion (ie, performance, mean step length and associated variability, and mean walking speed and associated variability), further levels of analyses (ie, intertrial and trial-by-trial analyses) will investigate the onset of the regulation, the regulation strategy, the relationship between needed and produced regulations over the obstacle approach, and the strength of the perceptual motor coupling. Our hypothesis was that the combination of traditional measures and these new measures should provide a more integrated understanding of the improvements caused by the implementation of VR training protocols. While traditional measurements are focused on the behavior of the older adult, the new measurements make it possible to analyze their behavior in relation to the environment. We believe that the joint use of these levels of analysis allows a finer characterization of product behavior and its evolution.

Finally, in the mixed condition, a gait adaptability score will be calculated based on gait performance and speed. The gait adaptability score is calculated from the pointing, crossing, and stepping performances modulated by the gait speed. The score is the product of gait performance and velocity so that it accounts for the capacity of older adults to manage the speed-accuracy conflict in the context of producing a goal-directed locomotor displacement. The performance obtained is the sum of the performance achieved in the two tasks. In the pointing task, performance is higher the closer the foot is to the mark (a score of 10 when the tip of the foot is <10 cm from the edge of the target, 8 between 10 and 15 cm, etc). In stepping-over tasks, performance is maximum (a score of 10) when the task is carried out in accordance with the instructions (ie, without meeting the obstacle), while imperfect completion of the task gives rise to a score of 5. The same methodology is applied during the crossing task. The sum of the performances achieved gives rise to an overall performance for each trial (maximum 20) that will be weighted by the average walking speed to complete the route (eg, 20×0.90=18).

#### VR-HMD Acceptance

For the VR group only, VR-HMD acceptance before and after use will be assessed using the TAM. According to this model, intention to use a technology is positively predicted by its perceived usefulness and its perceived ease of use. VR-HMD acceptance will be assessed through 4 variables: perceived usefulness, perceived ease of use, perceived enjoyment, and intention to use [54,73,74]. Participants will answer the 3 items per variable on a Likert scale from 1 (strongly disagree) to 10 (strongly agree): perceived usefulness (eg, “I believe using VR hardware would help me be more effective in my locomotion”), perceived ease of use (eg, “I believe using VR hardware would be easy for me”), perceived enjoyment (eg, “I believe I would find using VR hardware enjoyable”), and intention to use (eg, “I intend to use VR hardware within the foreseeable future”). In older adults, the TAM has been recently used to study VR-HMD acceptance before [44,53] and after the use [44] of a device intended to prevent falls among older adults.

### Secondary Outcome Variables

As mentioned in the Introduction section, interactions exist among gait behavior, functional capacities, and psychological states. In addition to the study of gait adaptability behavior and VR-HMD acceptance, several secondary variables will be investigated to characterize potential functional capacities and psychological state changes over the gait training programs.

#### Functional Capacities

Mobility evaluation will be assessed with the Timed Up and Go test [75]. Participants must sit down on a chair with their feet on the floor and their hands on their legs. At the “go” signal, they will stand up, walk 3 m, turn around the cone, walk back, and sit down again. The performance time (in seconds) will start when the participant starts moving, and it will end when the participant sits down again. Two trials at a self-paced velocity and 2 as fast as possible will be performed. For analysis, the mean value of the 2 trials in each condition will be considered.

Balance evaluation will be assessed with the Unipedal stance test [76]. Participants must lift one of their legs (while looking at a mark placed in front of them at eye height) and keep a stable equilibrium. The performance time (in seconds) will end when participants lose equilibrium (with a maximum trial duration of 60 seconds). Three trials with eyes open and 3 with eyes closed will be performed. For analysis, the best value of the trials in each condition will be considered as recommended in the study by Springer et al [76].

#### Psychological Assessment

Motivation for physical activity will be assessed using the motivation scale for physical activity for health purposes questionnaire recently validated in French [60]. This provides a measure of motivation to engage in physical activities from a health perspective through different behavioral regulations: intrinsic motivation, integrated regulation, identified, introjected, and extrinsic motivation, and amotivation. Intrinsic motivation will be assessed using the Inventory Motivation inventory questionnaire [61]. This provides a multidimensional measure of subjective experience while performing a given activity through interest, perceived competence, effort, and tension. Fear of falling will be assessed using the French version of the Modified-Falls Efficacy Scale [77]. This provides the level of concern about falling during activities inside and outside the home.

To sum up, primary outcome variables related to gait adaptability behavior and VR-HMD acceptance (for the VR group only) will be assessed at T1, T2, and T3 in the same way. Secondary outcome variables related to functional capacities and psychological states assessment will be assessed at T0, T1, and T2 in the same way. Adherence to programs will be measured at each training session. Occurrence of falls and near falls will be assessed during the follow-up period (ie, T3 at 12 months after completion of the training program) *via* a smartphone app (eg, the iFall app [78]). Table 1 provides an overview of the outcome measurements.

**Table 1:** Outcome measurements schedule.

	T0 (pretests)	Training sessions	T1 (posttests)	T2 (retention)	T3 (follow-up)
Primary outcome variable related to gait adaptability behavior					
Gait adaptability test	✓		✓	✓	
Primary outcome variable related to VR-HMD^a^ acceptance					
Acceptance	✓		✓	✓	
Secondary outcome variables related to functional capacities					
Timed Up and Go test	✓		✓	✓	
Unipedal balance test	✓		✓	✓	
Secondary outcome variables related to psychological assessment					
Motivation for health-related physical activity (EMAPS^b^)	✓		✓	✓	
Inventory Motivation inventory	✓		✓	✓	
French version of the Modified-Falls Efficacy Scale	✓		✓	✓	
Adherence to programs		✓			
Incidence of trips and falls					✓

^a^VR-HMD: virtual reality head-mounted display.

^b^EMAPS: Echelle de Motivation pour l’Activité Physique à des fins de Santé.

#### Sample Size

The sample size calculation was performed using G*Power (version 3.1.9.7). To detect relevant within-between-group differences, we calculated a sample size sufficient to detect small effect sizes. On the basis of values generally observed in studies conducted in the field of acceptability and acceptance of technologies in general (because no study similar to ours has yet been conducted), f=0.2, α=.05, and *P*=.80 were chosen to favor clinically significant effect sizes [79]. To test the main hypotheses of this study, 50 participants are required. To compensate for possible dropouts, 5 more participants will be included in each group. The 2 groups will be composed of 30 participants each.

#### Statistical Analysis

The statistical software JASP (version 0.17.1; 2023) will be used to run statistical analyses. The statistical level of significance was set at α=.05. Nonparametric tests were used when the distribution of the data was nonnormal. The statistical analysis will allow us to determine the effects of the two different training programs on the primary and secondary variables. The analysis will include participants who completed all the pretests and the posttests. Adherence to programs will be measured by the absence rate (in terms of %). Comparisons within and between groups before and after the intervention will be done using repeated measures ANOVA and Tukey tests as post hoc analyses. Effect sizes will be calculated using partial η^2^.

## Results

Data collection will begin in September 2025, and the first results are expected in December 2025.

## Discussion

This study aims to compare the effectiveness of 2 gait training programs intended to improve older adults’ gait adaptability behavior. More precisely, the aim is to evidence that VR is the more relevant tool to propose gait training programs for healthy older adults compared with a conventional training program such as NW training.

### Why Should the VR Gait Training Program Be More Effective?

It is expected that the 2 gait training programs will make it possible to improve adaptive locomotor skills. However, we expect that VR training will show larger benefits.

The expected superior outcome of VR gait training in comparison with NW training is supported by many studies. Levin [48] showed that VR can enhance gait adaptability training benefits by proposing motivational training content. Recently, Delgado and Der Ananian [80] reviewed the great potential of using VR in fall prevention. In an encouraging study, Lima Rebêlo et al [34] found a greater effectiveness of a gait training program using an immersive VR device (ie, VR-HMD) in comparison with conventional ones with regard to balance improvements and fall reduction in older adults with balance disorders.

Even if the results discussed in the previous section reveal the potential of VR tools, the use of VR in the context of training protocols for older adults is in no way a guarantee of success, and a certain number of preliminary steps have been carried out before being able to propose this research protocol. First, it seemed necessary to us to assess the validity of the VR device that we plan to use in the context of this study. The demonstration of the similarity of the regulation behavior produced in an equivalent locomotion task performed both in VR and real reality was a *sine qua non* for the proposal of a training program in this type of VR [44]. We also believe that the choices made as part of our VR training program, in particular basing the training sessions on the main principles of learning, could potentiate the effects usually associated with the use of VR.

As already mentioned in the first part of this paper, we hypothesize here that by adopting a systemic approach consisting of exposing older adults to accident-prone situations systematically requiring the production of adaptive behavior, we endow them with the perceptual motor mechanisms best able to allow them to move in complete safety. The idea is to allow the older adults to mobilize their resources (perceptual, decisional, and motor) in an appropriate way to produce a behavior appropriate to the constraints encountered or even to develop a form of adaptive flexibility.

Finally, with greater individualization compared with the NW training and the possibility of offering feedback during training, the VR training should maximize the behavioral and psychological benefits of the training program in older adults.

### VR-HMD Acceptance

In accordance with previous studies, we expect that older adults will accept the VR-HMD before its first use [53] and also after use [44,81]. Moreover, we expect that acceptance will increase at the beginning of the gait training with an augmentation of both perceived usefulness, perceived enjoyment, and perceived ease of use. On the basis of the previous literature review focusing on the TAM, we know that intention to use a VR-HMD is positively predicted by 3 variables: perceived usefulness, perceived enjoyment, and perceived ease of use. Due to motivating training content leading to high adherence, perceived ease of use and perceived enjoyment should be superior to the mean of the scale during the VR gait training and remain constant until the end. In addition, perceived usefulness should also be superior to the mean of the scale because the training program is designed to develop gait adaptability behavior. By confronting older adults with several goal-directed tasks, they should have a positive perception of device utility during the VR gait training.

### Differences in Effectiveness on Secondary Variables

In terms of functional capacities and psychological variables, both gait training programs should lead to improvements. Physical activity adopting a systemic approach provides beneficial effects on physical and psychological components, which are linked to gait behavior and thus to fall prevention [20,64]. However, due to VR’s large potentiality in gait training program implementation, improvements after the VR training (and long-term effects) should be greater on these general health components.

### Limitations of This Study

This research protocol was designed to overcome some conceptual and methodological limitations identified in previous studies. However, this protocol also has a certain number of limits linked, for example, to the choice of the population studied or to the possibilities of deploying VR training protocols in the clinical sector. Even if the choice of the population (healthy older adults) as part of the implementation of a preventive approach is totally committed, it is possible to question the possibility in the future of adapting the protocol to target other populations (older adults who fall or those with specific pathologies) within the framework of the implementation of a curative approach. On the material level, the device developed to carry out this study (Delbes et al [44] give a complete description) includes, among other elements, an aluminum structure permanently fixed and therefore not transportable, which limits for the moment the implementation of training protocols in other locations, which constitutes a limiting factor. Finally, although this measurement is not initially included in our protocol, a measurement of the VR device’s usability could also be considered later.

### Conclusions

The validation of the hypothesis concerning the greater effectiveness of the VR gait training program compared with the training program of NW should confirm that VR is the more promising tool in fall prevention. In addition, the study of VR-HMD acceptance in a longitudinal way (over a training program) should allow a better understanding of the psychological determinants of the behavioral intention to use technology among older adults to prevent falls. Increasing the effectiveness and acceptance of older adults’ training programs based on XR devices to prevent fall occurrence is a challenge to be met in the face of such a significant health issue.

## Abbreviations

AR: augmented reality
NW: Nordic walking
TAM: Technology Acceptance Model
VE: virtual environment
VR: virtual reality
VR-HMD: virtual reality head-mounted display
